# MicroRNA isolation and quantification in cerebrospinal fluid: A comparative methodical study

**DOI:** 10.1371/journal.pone.0208580

**Published:** 2018-12-07

**Authors:** Alena Kopkova, Jiri Sana, Pavel Fadrus, Tana Machackova, Marek Vecera, Vaclav Vybihal, Jaroslav Juracek, Petra Vychytilova-Faltejskova, Martin Smrcka, Ondrej Slaby

**Affiliations:** 1 Central European Institute of Technology (CEITEC), Masaryk University, Brno, Czech Republic; 2 Department of Comprehensive Cancer Care, Masaryk Memorial Cancer Institute, Faculty of Medicine, Masaryk University, Brno, Czech Republic; 3 Department of Neurosurgery, University Hospital Brno, Faculty of Medicine, Masaryk University, Brno, Czech Republic; University of Texas MD Anderson Cancer Center, UNITED STATES

## Abstract

Associated with the pathogenesis of many cancers, including brain tumors, microRNAs (miRNAs) present promising diagnostic biomarkers. These molecules have been also studied in cerebrospinal fluid (CSF), showing great potential as a diagnostic tool in patients with brain tumors. Even though there are some biological and technological factors that could affect the results and their biological and clinical interpretation, miRNA analysis in CSF is not fully standardized. This study aims to compare several RNA extraction and miRNA quantification approaches, including high-throughput technologies and individual miRNA detection methods, thereby contributing to the optimization and standardization of quantification of extracellular miRNAs in CSF. Such knowledge is essential for the potential use of miRNAs as diagnostic biomarkers in brain tumors.

## Introduction

Primary brain tumors and brain metastases annually affect close to 40 patients per 100,000 persons worldwide, a still growing incidence rate [1, 2]. Prognosis and therapy depend on the brain tumor type, so an early and accurate diagnosis can significantly affect the quality of life and the survival of patients. Unfortunately, a diagnosis of brain cancer is often limited by the localization and heterogeneity of the tumor tissue. Like in the other cancers, when tissue diagnosis is impossible because of the tissue’s localization or a lack of precision, liquid biopsies are promising diagnostic approaches. Specifically in brain cancers, frequent discussions have focused on the diagnostic utilization of cerebrospinal fluid (CSF). Bathing the central nervous system, CSF is in direct contact with all brain components, including neoplasms, and thus it is a source of many potential biomarkers [3].

Perspective CSF biomarkers seem to be circulating microRNAs (miRNAs). MiRNAs constitute a class of single-stranded non-coding RNAs, about 18–25 nucleotides in length, which post-transcriptionally regulate gene expression, thus being key players in the regulation of all cellular processes. Usually tissue-specific, miRNAs are involved in the pathogenesis of many diseases, including brain tumors [4]. Circulating miRNAs have been detected in almost all human body fluids, such as blood plasma and serum, urine, saliva, tears, and cerebrospinal fluid [5]. Moreover, levels of selected circulating miRNAs have been repeatedly described to be associated with specific tumors, grades, stages, prognosis, and therapy response in cancer patients. Interestingly, miRNAs are highly stable and resist extreme conditions, such as ribonuclease activity, repeated freezing and thawing, boiling, low and high pH, and long-term storage at room temperature [5]. Recent studies have shown that deregulated levels of CSF miRNAs are associated with malignant tumors of CNS [6–9]. Qu *et al*. have also showed that miR-21 level in CSF enabled to identify glioma patients with higher sensitivity and specificity in comparison with the plasma/serum miR-21 level [10]. Taken together, analysis of circulating miRNAs in CSF seems to be a promising tool leading to the refinement of current brain tumor diagnostics [11, 12]. Unfortunately, such analysis in human body fluids can be affected by many biological and technological factors, thus posing quite a challenge. Here we compare several approaches and protocols for RNA extraction from CSF and for conducting high-throughput and individual miRNA analyses in CSF.

## Material and methods

### Clinical samples

In this study, we included CSF samples collected from 10 glioblastoma (GBM) patients and 10 non-tumor donors (patients with hydrocephalus). From each person, a sample of 3–5 ml of CSF was collected through lumbar puncture (between the L3 and L5 vertebraes). The samples were taken in 2016 at the Department of Neurosurgery, The University Hospital Brno (Brno, the Czech Republic). CSF samples containing blood cells were excluded. All the patients signed informed consents for the use of CSF and clinical data for research purposes. The study was approved by the local Ethics Committee at The University Hospital Brno.

### CSF handling and sample preparation

CSF samples were centrifuged at 500g for 10 min at 4°C (Eppendorf 5810 R, Germany), and the supernatant were aliquoted to 1 ml tubes and stored at -80°C till further analyzed. For the first step of this study, that is, to select the most efficient method for RNA extraction from CSF, two GBM and two control independent CSF pools were prepared, each pool made from five different CSF samples. To prevent repeated thawing, the pools were aliquoted into tubes according to the volumes required for particular RNA isolations.

### RNA isolations

**Fig 1:**
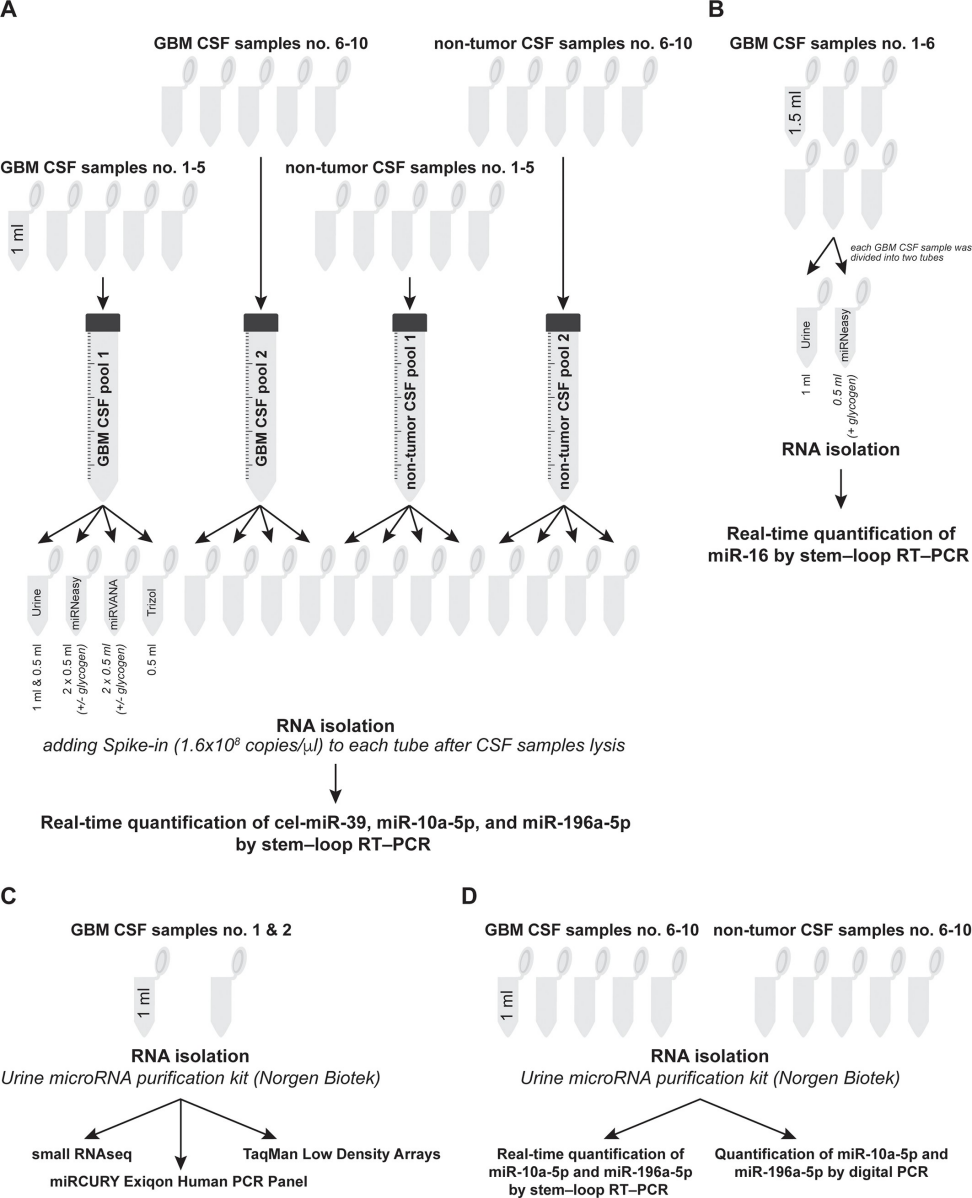
An illustrated workflow of RNA extraction optimization (A, B), high-throughput miRNA analysis (C), and the selection of miRNA analysis (D) methods.

We compared four different commercially available RNA purification kits: Urine microRNA purification kit (Norgen Biotek, Canada), miRNeasy Serum/Plasma kit (Qiagen, Germany), miRVANA miRNA Isolation Kit (Ambion, Austin, TX, USA), and Trizol reagent (Thermo Fisher Scientific, USA). The manufacturers’ protocols were followed except for the following modifications: In the case of Norgen, both 1 ml and 0.5 ml of CSF were used as an input volume for RNA extraction, and in the case of Qiagen and Ambion, 50 ng of glycogen (a co-precipitant and carrier) per isolation was added or omitted. At the elution step, samples were incubated on the column for 20 min at RT (except for the miRVANA kit, in which the elution buffer is heated to 96°C); the volume of elution buffer was in all cases modified to 30 μl. After adding of lysis buffer, 3.5 μl of miRNeasy Serum/Plasma Spike-In Control (1.6 × 10^8^ copies/μl, C. elegans miR-39 miRNA mimic, Qiagen, Germany) were added to each sample and mixed thoroughly. The spike-in controls were diluted according to the manufacturers’ protocols. Fig 1A and 1B show the workflow of CSF RNA extraction optimization.

### miRNA expression analysis

#### Spike-in detection

The RNA samples supplemented with miRNeasy Serum/Plasma Spike-In Control were transcribed using miScript II RT Kit (Qiagen, Germany), according to the manufacturer’s protocol. Real-time PCR was performed by miScript SYBR Green PCR Kit and Cel_miR-39_1 miScript Primer Assay (both Qiagen, Germany), using LightCycler 480 Instrument II (Roche, Switzerland), according to Qiagen’s protocol.

#### RT-qPCR and digital PCR

Reverse transcription was performed by TaqMan Reverse Transcription Kit (Applied Biosystems, USA) with stem-loop miRNA specific primers (hsa-miR-10a-5p; ID 000387 and hsa-miR-196a-5p; ID 241070 and miR-16; ID 00391). Real-Time PCR was performed using TaqMan Universal PCR Master Mix, NoUmpErase UNG (Applied Biosystems) and LightCycler 480 Instrument II (Roche, Switzerland). All steps were performed according to the TaqMan MicroRNA Assay protocol (Applied Biosystems, Foster City, CA, USA). Data were analyzed using the Fit point method of the LightCycler quantification software.

Digital PCR (dPCR) was performed using QuantStudio 3D Digital PCR 20K Chip Kit + Master mix v1 on a QuantStudio 3D Digital PCR Instrument (all ThermoFisher Scientific, USA), according to the manufacturer’s protocol.

#### High-throughput profiling methods

MiRCURY Exiqon Human PCR Panel I (miRCURY LNA, Exiqon, Denmark) was applied after Exiqon’s standardized protocol, using ExiLENT SYBR Green master mix, miRCURY LNA, microRNA Ready-to-Use PCR, Human Panel I, V4-R, Universal cDNA Synthesis Kit II, 8–64 rxns, miRCURY LNA, UniSp6 RNA spike-in control primer set v2 (all Exiqon, Denmark), and LightCycler 480 Instrument II (Roche, Switzerland).

TaqMan Low Density Arrays (Applied Biosystems, USA) were performed with the preamplification step, according to Applied Biosystems’s protocol, using TaqMan MicroRNA Reverse Transcription Kit, Megaplex RT Primers, Megaplex PreAmp Primers, Human Pool Set v3.0, TaqMan PreAmp Master Mix, TaqMan Array Human MicroRNA A+B Cards Set v3.0 and TaqMan Universal PCR Master Mix, No AmpErase UNG, 2x (all Applied Bioosystems, USA)

Next generation sequencing was performed using the NextSeq 500 (Illumina, USA) technology, with CleanTag Small RNA Library Preparation Kit (TriLink, Biotechnologies, L-3206) applied for library preparation and NextSeq 500/550 High Output Kit v2, 75 cycles (Illumina, USA) applied for sequencing run, according to the manufacturer’s protocol.

#### Data normalization

All real-time PCR reactions were run in triplicate, and for each sample the average threshold cycle and standard deviations (SDs) were calculated. Ct values were transformed using the 2^-ΔCt^ method (ΔCt = Ct(miRNA)– 40). The Wilcoxon pair test and the t-test were used to compare the efficiencies of the extraction methods. Spearman correlation was used to analyze relationships between the miRNA quantification approaches. All the analyses were performed using GraphPad Prism version 6.00 (GraphPad Software, San Diego, CA, USA), with the significance level of 0.05.

## Results

### Comparison of RNA extraction kits for miRNA isolation from cerebrospinal fluid

**Fig 2:**
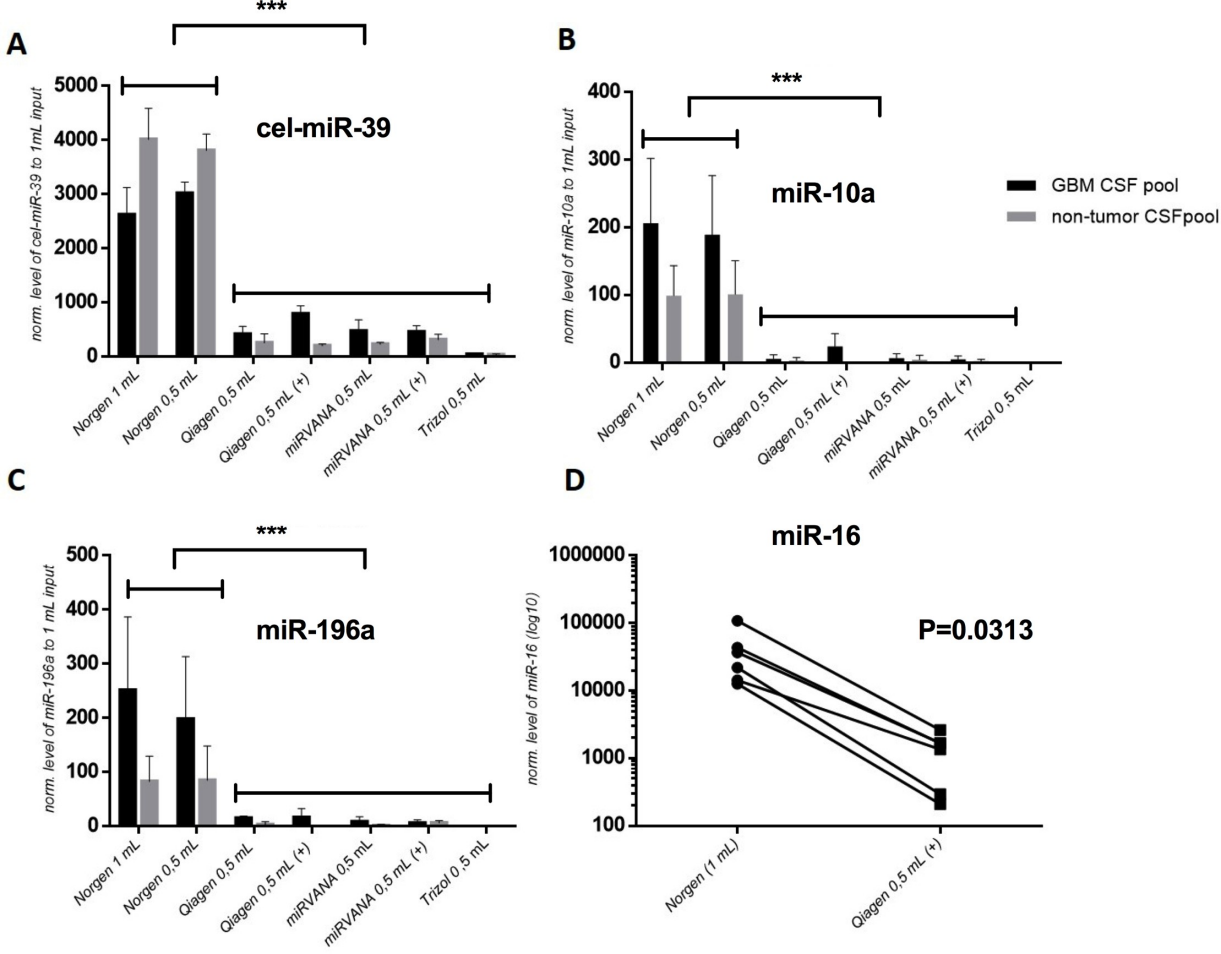
A comparison of selected CSF miRNA levels in RNA samples extracted by four RNA isolation kits with various protocol modifications, including different volumes of CSF input (1 ml and 0.5 ml) and adding (+) or omitting of glycogen during extraction. Levels of cel-miR-39 (A), miR-10a (B), and miR-196a (C) were analyzed using Real-Time PCR in RNA samples extracted from two GBM and two non-tumor CSF pools. Levels of miR-16 (D) were analyzed in paired RNA samples extracted from six independent CSF samples.

Using the four commercially available RNA extraction kits, we obtained very low RNA concetrations/yields from CSF—too low to use them for their comparison. Therefore, to evaluate the efficiency of miRNA extraction, we used real-time PCR quantification of cel-miR-39 spike-in control and two endogenous miRNAs (miR-10a-5p and miR-196a-5p). We chose these two particular miRNAs after a preliminary experiment, in which they showed significantly higher levels in GBM CSF samples compared to non-tumor CSF samples (data not shown). The highest levels of the miRNAs analyzed were detected in the RNA samples extracted using the Norgen kit (p < 0.001). The examined miRNA levels did not significantly differ between RNA samples extracted from both 0.5 and 1 ml of CSF (Fig 2A, 2B and 2C). Moreover, miR-16-5p (which was selected based on a previous study, in which it had detectable levels in both glioma [Ct means 26.28] and control CSF samples [Ct means 28.69] [7]) was quantified in six RNA samples extracted from independent GBM CSFs the using the Norgen and Qiagen kits supplemented with glycogen. CSF RNA samples extracted using the former kit showed significantly higher levels of miR-16 than those extracted using the latter kit (p = 0.0313 in the Wilcoxon pair test, Fig 1D).

### Comparison of high-throughput technologies for miRNA profiling in CSF

**Fig 3:**
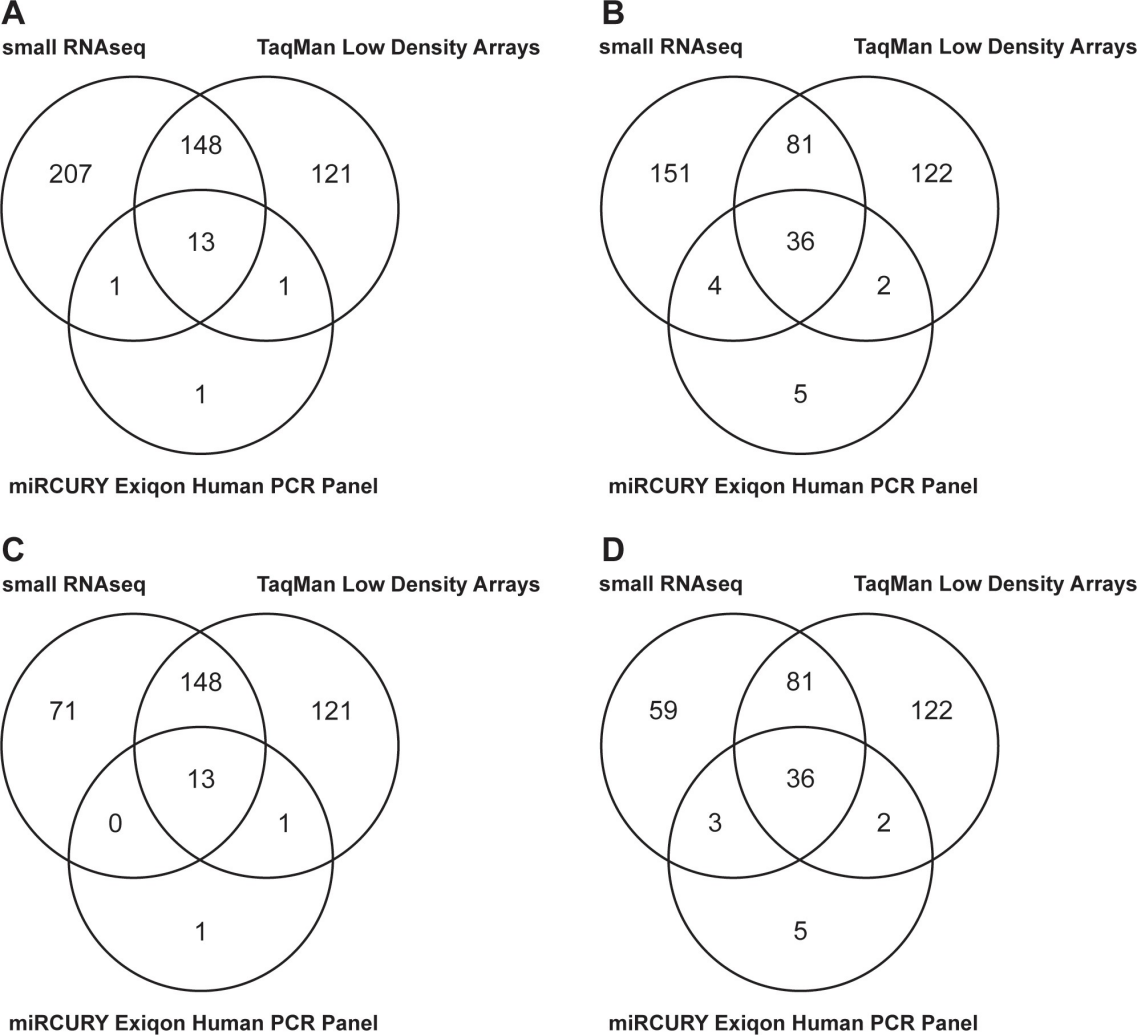
Venn diagrams showing an overlapping of detected miRNAs between different high throughput technologies, applying all the detected miRNAs in CSF sample A (A) and CSF sample B (B), and applying only a set of TLDA predesigned miRNAs in CSF sample A (C) and CSF sample B (D).

The NGS-based technology detected the most miRNAs in both analyzed samples: 369 (median of 31) and 272 (median of 18) miRNAs with at least one raw read per sample (Table 1, Fig 1C). Between the two examined real-time–PCR–based methods, TaqMan Low Density Arrays (TLDA; Thermofisher Scientific) with a preamplification step was more effective, with 283 (median Ct value of the detected miRNAs of 29.7) and 241 (median of 30.8) detected miRNAs of the 754 pre-designed miRNAs. The Exiqon technology without a preamplification step detected only 16 (median Ct value of the detected miRNAs of 33.7) and 47 (median of 33.3) of the 372 pre-designed miRNAs. S1 Table lists miRNAs detected with at least two of the above technologies. Venn diagrams (Fig 3A, 3B, 3C and 3D) show the numbers of miRNAs detected by the high-throughput technologies compared. Small RNAseq analysis detected most individual miRNAs (Fig 3A and 3B), a number dramatically reduced when comparing only miRNAs pre-designed in TLDA (Fig 3C and 3D).

**Table 1:** A comparison of the selected high-throughput technologies for miRNA profiling in cerebrospinal fluid and the number and quantity of miRNAs detected in the study.

Method	NGS		TLDA (A+B Card) with pre-amplification		miRCURY LNA (Panel I) without pre-amplification	
Sample	Sample A	Sample B	Sample A	Sample B	Sample A	Sample B
The number of possibly detected miRNAs	unlimited		754		372	
The number of detected miRNAs	369^§^	272^§^	283^#^	241^#^	16^#^	47^#^
Median of reads or Ct values of detected miRNAs*	31 (12/137)	18 (6/93)	29.7 (26.9/32.4)	30.8 (27.6/32.8)	33.7 (32.7/34.4)	33.3 (31.5/34.4)

^#^Ct < 35

^*^25/75% percentiles of the number of detected miRNAs

^§^ number of raw reads ≥ 1

**Fig 4:**
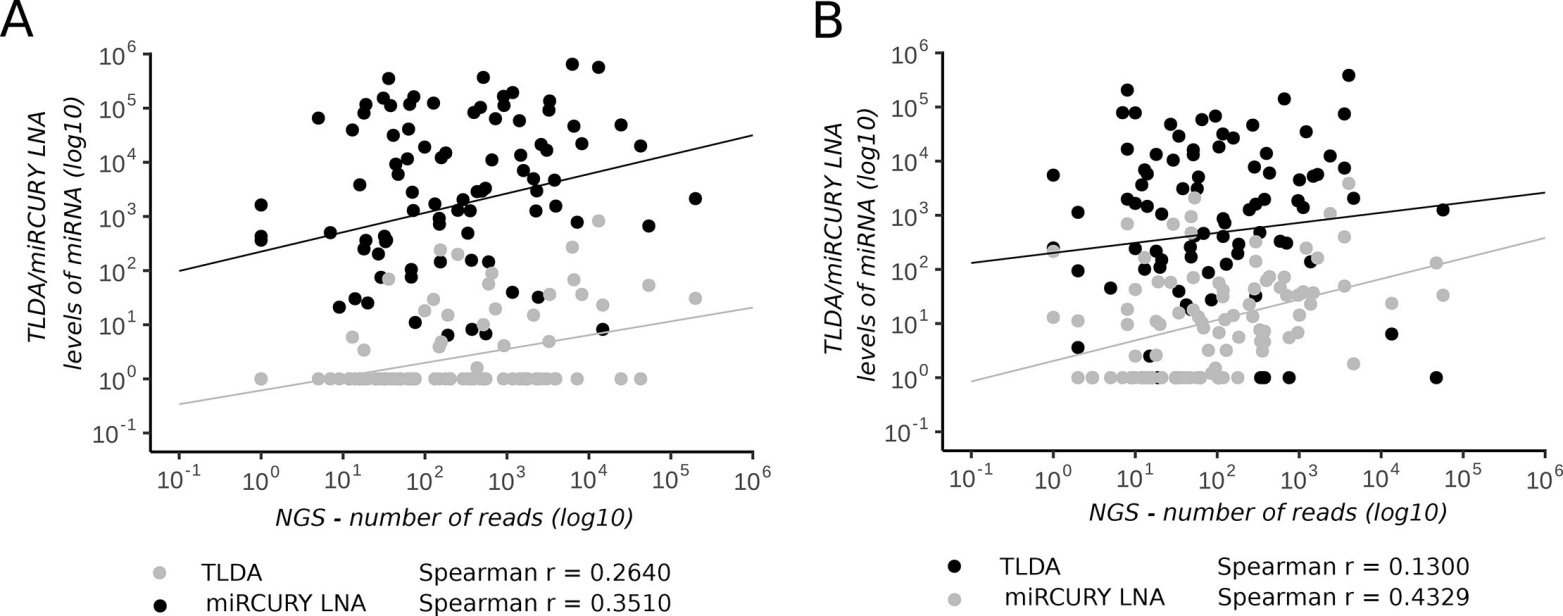
Correlation analyses of miRNA levels detected using the Exiqon and TLDA pproaches and the NGS platform in (A) CSF sample A and (B) CSF sample B.

The results of the PCR-based technologies and those of the NGS platform were only weakly correlated in both samples examined (Fig 4). Specifically, correlation coefficients between NGS platform and Exiqon technology reached 0.35 and 0.43 in samples A and B, and those between the NGS platform and the TLDA method reached 0.26 and 0.13, respectively.

### Comparison of real-time PCR and digital PCR technologies for quantification of individual miRNAs in CSF

**Fig 5:**
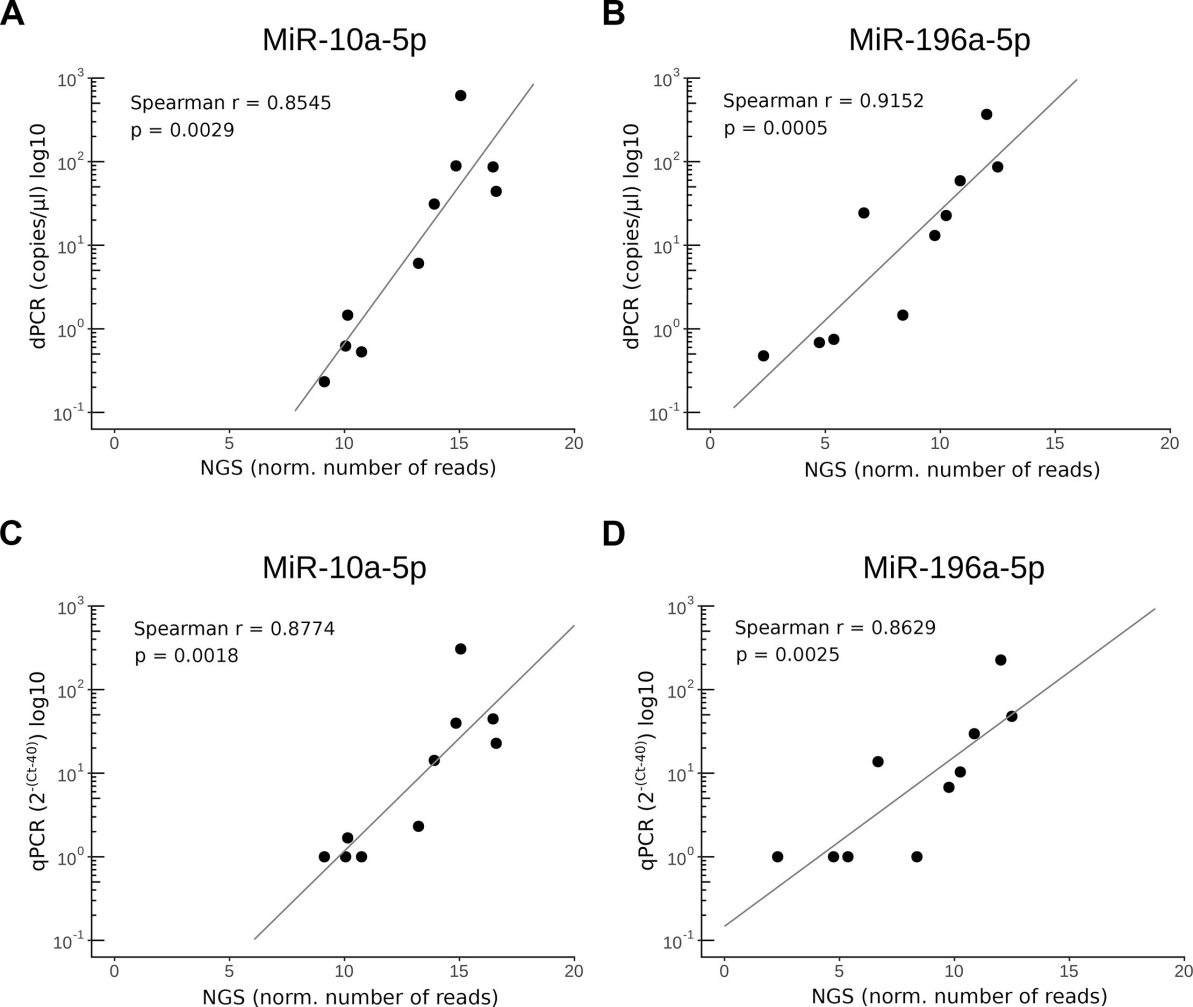
Correlation analyses of miR-10a-5p and miR-196a-5p levels detected using (A,B) digital PCR and (C,D) real-time PCR technologies and NGS platform in CSF samples.

Based on our previous experiences with these miRNAs, we selected miR-10a-5p and miR-196a-5p for quantification using the real-time PCR and digital PCR technologies. These analyses were performed in CSF samples collected from five patients with primary GBM and five healthy donors (Fig 1D). According to Spearman correlation, the results of the PCR-based technologies and the NGS platform were highly correlated. Specifically, the correlation between digital PCR and NGS reached 0.85 in miR-10a-5p and 0.92 in miR-196a-5p (Fig 5A and 5B). Similar correlation was observed between real-time PCR and NGS (r = 0.88 in miR-10a-5p and 0.86 in miR-196a-5p; Fig 5C and 5D).

## Discussion

To find RNA extraction method providing the highest miRNA levels from CSF samples, we compared four commercially available RNA isolation kits, following the recommended protocol and with its small modifications related to glycogen supplementation, CSF input volumes, and time of RNA elution [13, 14]. Unfortunately, the RNA yields obtained were undetectable by common fluorospectrophotometer-based methods, such as the Nanodrop and Qubit technologies (both ThermoFisher).

Therefore, using the real-time PCR technology to evaluate extracted miRNA levels, we quantified three endogenous miRNAs (miR-16, miR-10a-5p, and miR-196a-5p) and exogenous cel-miR-39, which were added during RNA extraction process. To avoid cel-miR-39 degradation by endogenous RNases, we added it into the sample after the lysis step [15]. In our hands, Urine microRNA Purification kit from Norgen, which eventually eluted into 30 μl of elution buffer after 20 minutes of incubation on the column, showed the highest cel-miR-39 recovery rate. The same extraction protocol lad to the highest levels of all the endogenous miRNAs analyzed. Based on these results, we suggest Urine microRNA Purification kit from Norgen to be the most appropriate for miRNA extraction from CSF samples. Thus, we used this kit for RNA extraction in the following analyses. However, levels of spike-in cel-miR-39 varied between the pools. This may be due at least in part to the fact that we made a new dilutions of spike-in from a highly concentrated stock several times during the study. When the most efficient RNA isolation approach (Norgen) was used, the differences between pools ranged from 25–30%, which when expressed in Ct values means dCt less than 0.4 between pools. It should be recommended to prepare and use only the one dilution of spike-in cel-miR-39 for the whole experiment to eliminate technological variability in its quantification.

The potential of CSF miRNAs to serve as the accurate brain tumor biomarkers depends on methodological approaches used for their quantification. Unfortunately, methods commonly used for high-throughput miRNA profiling require a higher RNA input than RNA yields recovered from CSF samples. Moreover, these methods are optimized for RNA specimens extracted from cells and tissues. A better option is the manufacturing protocol supports RNA extracted from blood plasma/serum samples. However, there is no commercially available method for CSF miRNA quantification. In this regard, miRNA profiles in cell/tissue, blood plasma/serum, and CSF samples show significantly different patterns. Specifically, Iwuchukwu *et al*. analyzed 782 known miRNAs (Exiqon) in plasma and CSF samples and identified significantly more miRNAs in CSF than plasma [16]. Sorensen *et al*. [17] reported similar results. Akers *et al*. [18] found more specific miRNAs in glioblastoma tissue than in CSF [18].

Different distributions and proportions of miRNAs in total RNA yields can affect the accuracy of miRNA analysis. For high-throughput miRNA analysis in CSF samples, we compared two real-time–PCR–based technologies and an NGS platform in two independent CSF samples collected from GBM patients. NGS detected the most miRNAs. The PCR methods were more limited by the low RNA input because the number and proportion of detected miRNAs increased rapidly when a preamplification step was included. On the other hand, correlation analysis of miRNA levels detected using all three high-throughput approaches showed lower correlation between NGS and PCR with a preamplification step than that without it. Thus, it seems that a preamplification step preceding the final real-time PCR analysis biased the results. Since NGS is able to detect not only miRNAs but also other small RNA classes (including PIWI-interacting RNAs [19]) and to determine their isoforms [20], we suggest that a NGS platform is the most suitable for the analysis and quantification of miRNAs in CSF samples. The feasibility of this method for miRNA analysis in CSF samples was previously examined and confirmed by Burgos *et al*. [13].

We compared real-time PCR with digital PCR. Although real-time PCR is nowadays the most established method of miRNA expression analysis, it has some limitations. Its main weaknesses are low sensitivity and accuracy in low-copy template detection [21] and complicated raw data normalization (especially in body fluids), all of which can bias final results. On the other hand, Conte *et al*. showed dPCR to be accurate, reproducible, and reliable—and thus more appropriate for the identification and quantification of miRNAs in body fluids [22]. In this study, we have compared miR-10a-5p and miR-196a-5p levels detected by real-time PCR and dPCR with NGS data in ten independent CSF samples. Although the results of the methods were highly correlated, our data suggest that real-time PCR is not able to precisely distinguish samples with lower than ten miRNA copies. Based on our results and those published in other studies, we suggest that dPCR is a more suitable method for the quantification of individual miRNAs in CSF samples.

There is no consensus on the best normalization approach, a challenging issue since analysis of miRNA levels in body fluids is affected by many technical and biological factors. In CSF samples, several methods have already been suggested, including exogenous spike-in miRNAs, reference endogenous small RNAs, and global mean normalization approaches used in high-throughput analyses. However, all these methods have limitations. Spike-in miRNAs do not reflect biological factors. Endogenous small RNAs that are stably expressed in cells—such as RNU 44, RNU 48, and RNU 6B—show varying levels in individual CSF samples. Moreover, they do not fully reflect the biogenesis of circulating miRNAs [18]. Despite this, recent studies have suggested some promising reference circulating miRNAs (miR-24, miR-125, let-7c, miR-21, miR-24, miR-99b, miR-328 and miR-1274B, miR-15a-5p, miR-21-5p, miR-23a-3p, miR-23b-3p, miR-99a-5p, miR-125b-5p, miR-145-5p, miR-204-5p, and miR-320a) [7, 8, 23, 24]. Even though another study has already disproved some of these miRNAs as useful reference molecules for the normalization of CSF miRNA levels [25], using them remains a promising approach to objectivize results of CSF miRNA analysis.

## Conclusion

Circulating CSF miRNAs seem to be promising biomarkers that could help to refine current brain tumor diagnostics. However, analysis of these small non-coding RNAs in CSF is still not fully standardized, and many factors can bias the results. Thus, optimization and standardization of individual steps in this analytical process could bring CSF miRNAs closer to clinical use. After comparing several RNA extraction methods, we suggest that the Urine microRNA purification kit provided by Norgen Biotek company is the most appropriate kit for miRNAs extraction from CSF samples. Further, our data show small RNAseq and digital PCR to be suitable methods for CSF miRNA quantifications.

## Supporting information

S1 Table MicroRNAs d with at least two of the tested high-throughput technologies. (XLSX)
